# How Do Parameters of Implant Primary Stability Correspond with CT-Evaluated Bone Quality in the Posterior Maxilla? A Correlation Analysis

**DOI:** 10.3390/ma14020270

**Published:** 2021-01-07

**Authors:** Ji-Hyun Kim, Young-Jun Lim, Bongju Kim, Jungwon Lee

**Affiliations:** 1 Department of Prosthodontics and Dental Research Institute, School of Dentistry, Seoul National University, Seoul 03080, Korea; kjhlot@naver.com; 2 Dental Life Science Research Institute & Clinical Translational Research Center for Dental Science, Seoul National University Dental Hospital, Seoul 03080, Korea; bjkim016@gmail.com; 3 Department of Periodontics, One-Stop Specialty Center, Seoul National University, Dental Hospital, Seoul 03080, Korea; jungwonlee.snudh@gmail.com

**Keywords:** bone density, Hounsfield units, insertion torque, resonance frequency analysis, posterior maxilla

## Abstract

The aim of the present study was to evaluate correlations between bone density and implant primary stability, considering various determinants such as age, gender, and geometry of implants (design, diameter). Bone density of edentulous posterior maxillae was assessed by computed tomography (CT)-derived Hounsfield units, and implant primary stability values were measured with insertion torque and resonance frequency analysis (RFA). A total of 60 implants in 30 partially edentulous patients were evaluated in the posterior maxilla with two different types of dental implants. The bone density evaluated by CT-derived Hounsfield units showed a significant correlation with primary stability parameters. The bone quality was more influenced by gender rather than age, and the type of implant was insignificant when determining primary stability. Such results imply that primary stability parameters can be used for objective assessment of bone quality, allowing surgical modifications especially in sites suspected of poor bone quality.

## 1. Introduction 

A successful implant depends on patient-related (e.g., bone volume and density) and procedure-related parameters (e.g., design, diameter and length of implant, surgical procedure). There can be flexibility in implant design and surgical techniques, although some parameters such as bone density cannot be modified by the operator [1].

The term “bone quality” encompasses many broad concepts of bone including physiology, mineralization, and morphology [2]. According to the classification suggested by Lekholm and Zarb, bone density can be classified into four types based on the amount of cortical versus cancellous bone in the alveolar bone examined on pantograph film [3]. Misch further characterized the four bone density classes based on the tactile sense of the clinician placing the implant [4]. However, a distinction between the four types of bone has not been clearly established.

Computed tomography (CT) is useful when assessing the relative distribution of compact and cancellous bone. Bone density can be evaluated using Hounsfield units (HU) in CT, which are expressed by CT attenuation values according to a linear density scale [5]. The Hounsfield scale is used to evaluate bone for implant placement, and these values were considered site specific, objective, and quantitative. HU value is related to the density of the tissue represented by the voxel bone density classification, which is categorized as follows: D1, >1250 HU; D2, 850–1250 HU; D3, 350–850 HU; D4, 150–350 HU [6].

The poorest intraoral bone quality is typically found in the posterior maxilla. Shapurian et al. reported that more than 80% of the edentulous posterior maxillae consisted of porous cortical crest or no cortical bone [5]. Although most of the posterior maxillae were classified as D3 or D4, there were remarkable variations among individuals.

Generally, bone quality is considered the primary cause of different survival rates examined in the maxilla and mandible. Modifications in implant design, implant number, and surgical techniques [7] are required to better suit implant surgery in D4 bone. In an analysis of 3937 patients who had received a total of 12,465 dental implants, Goiato et al. reported implant survival rates according to bone density: type I, 97.6%; type II, 96.2%; type III, 96.5%; and type IV, 88.8% [8].

Primary stability of implants is commonly considered as a key factor for achieving successful osteointegration [9]. Primary stability is influenced by various factors, such as the length and diameter of the implant, its design, the micro-morphology of the implant surface, the insertion technique and the congruity between the implant and the surrounding bone [10]. Further important determinants are the quality and quantity of the bone at implant sites [11].

Several methods can be used to measure primary implant stability. Insertion torque (IT) measurement is one of the most commonly used methods. A high insertion torque value implies sufficient primary stability of implants while a low value indicates low primary stability with greater possibility of early failure [12]. As such, insertion torque measurement can be utilized in estimating the period with an optimal healed state suitable for a further load [13].

Non-invasive resonance frequency analysis (RFA) is another method used to measure primary implant stability [14]. Resonance frequency analysis makes it possible to determine primary stability immediately after placement, as well as secondary fixation after a period of healing. A metal rod (a peg) is connected to the implant by means of a screw connection. The peg is excited by magnetic pulses and the resonance frequency is expressed electromagnetically as an implant stability quotient (ISQ) with units ranging from 1 to 100, with higher values of the ISQ indicating higher implant stability [15].

The aim of the present study was to evaluate correlations between bone density and implant primary stability, considering various determinants such as age, gender, and geometry of implants (design, diameter). Bone density of edentulous posterior maxillae was assessed by computed tomography (CT)-derived Hounsfield units, and implant primary stability values were measured with insertion torque and resonance frequency analysis (RFA).

## 2. Materials and Methods

### 2.1. Patients and Implants

Patients who had undergone implant surgery between May 2012 and March 2015 in Seoul National University Dental Hospital were recruited in this retrospective study.

The study protocol was reviewed and approved by the Institutional Review Board of Seoul National University Dental Hospital (IRB No. CDE12001). All participants were informed about the nature of the study and signed the informed consent form.

A total of 60 implants in 30 patients (male: 20, female: 10) were included in this study. The participants had to have two consecutive unilateral losses in posterior maxillae with the intact occlusal plane opposed to the edentulous surgical site. The inclusion criteria consisted of an age of 18 years or older, residual bone height of 6 mm or more, and sufficient availability of alveolar bone in the surgical site. The exclusion criteria were general contraindications to implant treatment, recent tooth extraction or periodontal diseases surrounding surgical sites, residual width less than 1 mm after implant insertion, and bone graft materials more than 5 mm.

Preoperative examination was done via panoramic radiograph, computerized tomography (CT) scan, intraoral examination, and diagnostic casts. Two different types of implants were used in this study: SLActive^®^ Bone level implant (Institut Straumann AG, Basel, Switzerland) and CMI IS-II active^®^ implant (Neobiotech Co., Seoul, Korea). Patients were randomly assigned to one of two implant groups using the random distribution table. The length and diameter of implants used were 10 mm and 4–5 mm, respectively.

### 2.2. CT Scans

The CT scans were taken by a Somatom Sensation 10R multidetector system (Siemens AG, Erlangen, Germany) with the following parameters: 1 mm slice thickness; TI 0.75 s; 120 kV; and 150 mA/s. OnDemand^®^ software version 1.0 (Cybermed, Seoul, Korea) was used to plot the implants on CT scans. Using this software, the three-dimensional information from the post-operative CT image was compared with the pre-operative CT image, and the average bone density values of the internal and external part of implant were measured in HU. CT values obtained for the voxel labeling were limited to the rectangular area. The voxel values were computed in tens within the range of 150 to 2000 HU.

### 2.3. Surgical Procedure

Each participant received two implants from the assigned implant system. All implant sockets in the posterior maxilla were prepared according to the manufacturer’s instructions using a surgical micromotor by a single clinician. Implants were placed using single-stage surgery. The surgical procedure was performed under local anesthesia after antimicrobial prophylaxis with 500 mg of amoxicillin. The implant sites were exposed through a crestal incision followed by mucoperiosteal flap elevation. In case maxillary sinus augmentation was needed, a synthetic bone graft material (Calpore, Neobiotech Co., Seoul, Korea) was placed at 5 mm or less in height using a crestal approach. Healing abutments were attached, and the soft tissue was sutured in place.

### 2.4. Insertion Torque Measurement

The maximum IT value of each implant was recorded with INTRAsurg 300 (Kavo Dental Ltd., Amersham, UK). The IT was increased from 20 Ncm in 5-Ncm increments until the operator was unable to rotate the implant due to friction, before complete insertion of the implant. The insertion torque was aimed to be within 30–50 Ncm and was adjusted by using a larger implant or rotating the implant in the opposite direction if the value was out of the range.

### 2.5. Resonance Frequency Measurements

Evaluation of resonance frequency analysis (RFA) was performed immediately after implant placement using the Osstell device (Integration Diagnostics AB, Gothenburg, Sweden). The SmartPegs were mounted on the implants and manually screw-tightened. The RFA value was measured four times in each of the four directions (mesial, distal, buccal, and lingual) for each implant. RFA values are represented in the unit called the implant stability quotient (ISQ), which ranges from 1 to 100. A higher ISQ value indicates greater stability.

### 2.6. Statistical Analysis

The Pearson test was used to determine possible correlations between bone density, IT values, ISQ values to implant recipient regions, implant dimensions, and patient sex and age (older versus younger). The Student t test for independent variables was used for comparative analysis of different bone densities, IT values, and ISQ values based on age, sex, and treated arch. *p* values < 0.05 were considered to be significant.

## 3. Results

**Figure 1:**
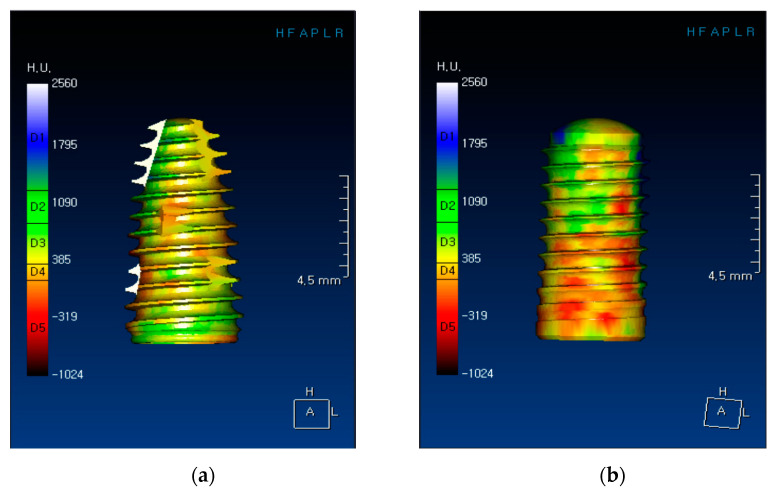
Three-dimensional images of implants showing bone types and corresponding Hounsfield units, generated by OnDemand^®^ software (Cybermed, Seoul, Korea): (**a**) CMI implant and (**b**) SLActive implant.

**Figure 2:**
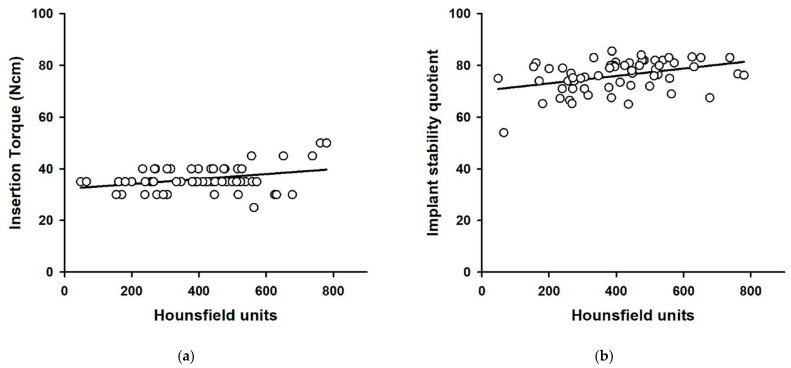
Scatter plots of Hounsfield units vs. insertion torque (**a**) and Hounsfield units vs. implant stability quotient (**b**). Solid lines show the regression line.

Figure 1 presents three-dimensional images of the implants, showing bone types and corresponding Hounsfield units generated by OnDemand^®^ software. Table 1 shows respective bone densities, insertion torque values, and RFA values to sex, age, implant type, and bone classification. The mean bone density, maximum insertion torque values, and mean resonance frequency analysis (RFA) values of all 60 implants were 404 ± 165 HU, 36.1 ± 4.9 Ncm and 76.0 ± 6.1. Bone density in the 30 patients ranged between 48 and 780 HU. Statistically significant relationships between bone density values and insertion torque measurements as well as between bone density and RFA values were commonly observed in all implant sites (*p* < 0.05) (Table 1 and Figure 2).

Table 2 presents bone density, insertion torque values, the RFA values, and corresponding correlations with respect to implant dimension. A statistically significant relationship between bone density values and diameter of implant (*p* < 0.01) was observed, but not between insertion torque values and RFA values.

**Figure 3:**
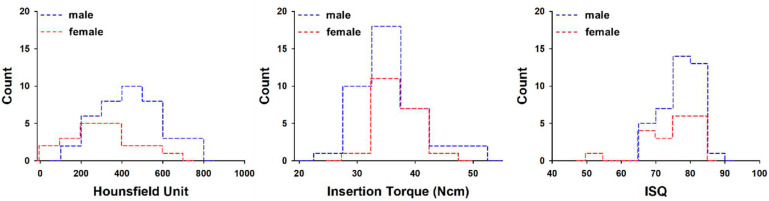
Histogram of average Hounsfield units, maximum insertion torque, and implant stability quotient values with patient gender. ISQ = implant stability quotient.

The mean bone density, insertion torque, and RFA values were 311 ± 158 HU, 37.0 ± 3.4 Ncm, 74.5 ± 7.5 for the 20 implants placed in female patients. The mean bone density, insertion torque, and RFA values for the 40 implants placed in male patients were 451 ± 150 HU, 35.6 ± 5.5 Ncm, and 76.8 ± 5.2. HU value differences between females and males were statistically significant (*p* < 0.001), but not in IT and RFA values (*p* > 0.05) (Figure 3).

**Figure 4:**
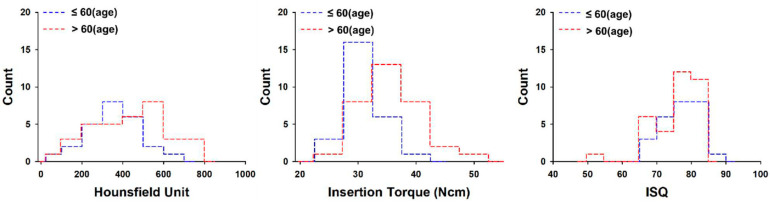
Histogram of average Hounsfield units, maximum insertion torque, and implant stability quotient values with patient age. ISQ = implant stability quotient.

The under-60 age group exhibited mean bone density, insertion torque, and RFA values of 362 ± 134 HU, 36.0 ± 3.5 Ncm, and 76.1 ± 5.6, respectively, for 26 implants, while respective values were 436 ± 181 HU, 36.2 ± 5.8 Ncm, and 76.0 ± 6.5 for 34 implants placed in the over-60 age group. The over-60 age group showed higher mean bone density values, but the difference between the under-60 and over-60 age groups was not statistically significant (*p* > 0.05) (Figure 4).

**Figure 5:**
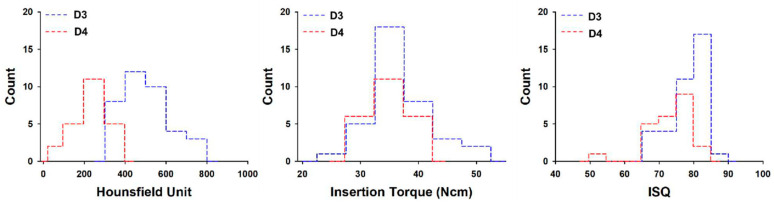
Histogram of average Hounsfield units, maximum insertion torque, and implant stability quotient values with type of bone. ISQ = implant stability quotient.

The bone quality of posterior maxillae in this study was classified as D3 (37 implants) or D4 (23 implants) according to Misch’s classification. The mean bone density, insertion torque, and RFA values were 508 ± 111 HU, 37.8 ± 5.4 Ncm, and 77.9 ± 5.1 for 37 implants placed in the D3 group, while corresponding values were 237 ± 78 HU, 35.0 ± 3.7 Ncm, and 72.9 ± 6.4 for 23 implants placed in D4 group. In the D3 group, the relationship between bone density values and insertion torque measurements was statistically more significant than it was in the D4 group (*p* < 0.05). When compared to the D4 group (72.9 ± 6.4), significantly higher RFA values were found for the D3 group (77.9 ± 5.1, *p* < 0.05) (Figure 5).

**Figure 6:**
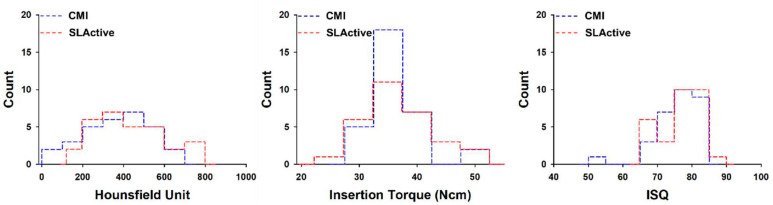
Histogram of average Hounsfield units, maximum insertion torque, and implant stability quotient values with type of implant system. ISQ = implant stability quotient.

Lastly, the mean bone density, insertion torque, and RFA values were 434 ± 1173 HU, 36.8 ± 6.1 Ncm, and 76.3 ± 5.9, respectively, for 30 implants placed in the SLActive group, while corresponding values were 374 ± 154 HU, 35.2 ± 3.2 Ncm, and 75.7 ± 6.4 for 30 implants placed in the CMI group. These two implant groups did not show any statistically significant differences in mean bone density, insertion torque, or RFA values (Figure 6).

## 4. Discussion

Bone density is a meaningful prognostic marker for evaluating the long-term success of implants [3]. The bone classification proposed by Lekholm and Zarb is now commonly used. However, this classification has its limitations in objectivity and reproducibility. In order to overcome the limitations of this classification, the evaluation of bone density using CT has been presented in several previous studies as an objective and reliable method.

The mean bone density value of the posterior maxillary region reported by Fuster-Torres et al. [2] was 464 HU for 25 implant sites, while Norton and Gamble [16] reported the mean bone density in 27 maxillary implant sites to be 417 HU. Turkilmaz et al. [17] and Shapurian et al. [5] performed similar studies, and their results were 403 HU for 70 implant sites and 333 HU for 54 implant sites, respectively. In this study, we obtained results similar to those reported by Turkilmaz et al. [17].

In addition, Turkilmaz et al. [18] and Isoda et al. [19] reported statistically significant correlations between HU and the parameters of primary stability, IT and RFA. These results were also found in our study. However, results of this study were different to those from previous studies, possibly due to different patient-related factors such as age and gender.

The bone quality of all posterior maxillae investigated in our study was classified as D3 or D4. Women exhibited lower HU values than men (*p* < 0.05) but there was no pronounced difference in HU values between groups over 60 and under 60. Turkilmaz et al. [17,18] demonstrated a statistically significant difference in mean bone density between men and women, whereas Shapurian et al. [5] found no difference. Our study results were consistent with the results of Turkilmaz et al. [17,18]. However, due to a lack of agreement among results from different experiments, further studies with a large sample of patients are necessary to gain a better understanding of the relationship between age, gender, and bone density.

Primary stability is a critical determinant in evaluating the success of immediate loading. Roccuzzo et al. [20] suggested that the minimum insertion torque values for unitary implants and multiple splinted implants be 30 and 20 Ncm, respectively, for immediate loading. In this study, the mean maximum IT value of 36.1 ± 4.9 Ncm and RFA value of 76.0 ± 6.1 were recorded, and no differences in bone density, age, and gender were observed.

In the CMI implant group, the reason for the HU-IT r value of 0.005 was that IT values were evenly distributed around 35 Ncm regardless of HU values, so that no correlation was found by the Pearson correlation test. On the other hand, in the SLActive implant group, when the HU has a high value over a certain level, the IT value tends to be high, so the HU-IT r value was analyzed as 0.462 (Table 1).

In the results of Table 2, almost constant IT and ISQ values were obtained after surgery, even though each implant site had different HU values, either high or low. The length of all implants investigated in this study was the same (10 mm), and there was a difference only in diameter. The results of this study suggest that changes in the diameter of the implant led to appropriate primary stability for immediate loading when bone density was poor. Therefore, it may be possible to overcome the risk of poor stability in areas of low-density bone through procedural techniques such as using an implant with a larger diameter.

The analysis according to gender shown in Figure 3 showed a difference only in HU values, and there was no difference in IT or ISQ. On the other hand, there was no difference in HU, IT, or ISQ between the over-60 and under-60 age groups (Figure 4). In general, as age increases, HU value is expected to be low; however, in our study, a lower HU value appeared only in young women under the age of 60, and these results eventually showed no difference in HU. The reason that there was no difference in the IT and ISQ values was that an implant with a large diameter was selected or a method such as under-drilling was used to obtain a primary stability for early loading during surgery.

Although there was a difference in HU and ISQ according to D3 and D4, there was no difference in IT, and there was no difference between both implant types (Figure 5 and Figure 6). It can be interpreted that the factor that affects primary stability during the implant procedure is more influenced by bone quality than implant type.

Regardless of implant systems, a strong correlation between HU and IT values was observed. However, when comparing different bone types, only the D3 group exhibited a correlation. A possible reason for this finding might be that the effect of HU on IT is offset by using an implant with a larger diameter in the D4 group. For IT and ISQ, the mean values in the D3 group were greater than those in the D4 group, but only the difference in the ISQ values gained statistical significance. Based on this result, it can be considered that bone quality affects the ISQ values more than the IT values.

In the present study, the parameters of primary stability of two types of implants exhibited no statistical significance. Our results were consistent with those reported by Rozè et al. [15], although different implant systems (Straumann and Ankylos) were used. O’sullivan et al. [21], in its investigation of primary stability of five types of implant systems with varying geometry and surface topography, concluded good primary stability was demonstrated in type 2 and 3 bone, although Mark IV implants appeared to also maintain a high primary stability in type 4 bone. However, such results must be approached carefully. Rabel et al. [4] suggest that ISQ values cannot be compared among different implant systems unless they are calibrated for each system separately. Although RFA values may be useful in determining different healing phases, they are not suitable for a standardized parameter to evaluate implant stability when used alone [2].

Determining factors of primary stability are macro-design features and micro-morphology of the implant, the insertion technique, and proximity between the implant and the surrounding bone [20]. However, previous studies have shown conflicting opinions regarding the influence of implant geometry on primary stability. While some authors concluded that the length and diameter of the implants do not significantly influence ISQ values [22,23], others demonstrated a positive correction between implant length or diameter and ISQ, particularly where poor bone quality was detected [24,25,26]. As a result, Barikani et al. [26] recommended performing bone augmentation in cases of low bone density and inadequate bone height, rather than utilizing short implants.

In short, our results demonstrated that just changing the diameter of the implant to a larger one was not effective enough to overcome poor bone quality and improve primary stability. Therefore, in order to obtain proper primary stability in such cases, clinicians should carefully plan the course of treatment and be cognizant of the future healing period, the loading protocols, and the prosthetic components.

## 5. Conclusions

It is important for the clinician to have adequate information about the bone quality of the prospective implantation site. Within the limitations of this study, it can be concluded that CT-derived HU is significantly correlated to the parameters of primary stability. The HU value was analyzed to be affected by the gender and type of bone (D3, D4), and the ISQ value was found to be affected only by the type of bone (D3, D4). These values can allow objective assessment of bone quality, resulting in modifications to a more appropriate surgical plan, especially in sites of poor bone quality.

## Figures and Tables

**Table 1:** Mean Hounsfield units, maximum insertion torque, and implant stability quotient values and corresponding correlations with sex, age, and types of implants and bone.

Group		Number of Patients	Number of Implants	Mean HU (±SD)	Mean IT (±SD)	Mean ISQ (±SD)	HU-IT r (*p*-Value)	HU-ISQ r (*p*-Value)
Total		30	60	404 ± 165	36.1 ± 4.9	76.0 ± 6.1	0.326 (0.011)	0.389 (0.002)
Sex	Male	20	40	451 ± 150	35.6 ± 5.5	76.8 ± 5.2	0.415 (0.008)	0.281 (0.079)
	Female	10	20	311 ± 158	37.0 ± 3.4	74.5 ± 7.5	0.474 (0.036)	0.457 (0.043)
Age	≤60	13	26	362 ± 134	36.0 ± 3.5	76.1 ± 5.6	0.407 (0.039)	0.417 (0.034)
	>60	17	34	436 ± 181	36.2 ± 5.8	76.0 ± 6.5	0.306 (0.079)	0.396 (0.020)
Type of implant	SLActive	15	30	434 ± 173	36.8 ± 6.1	76.3 ± 5.9	0.462 (0.010)	0.179 (0.343)
	CMI	15	30	374 ± 154	35.3 ± 3.2	75.7 ± 6.4	0.005 (0.981)	0.602 (0.001)
Type of bone	D3		37	508 ± 111	36.8 ± 5.4	77.9 ± 5.1	0.354 (0.032)	0.054 (0.750)
	D4		23	237 ± 78	35.0 ± 3.7	72.9 ± 6.4	0.178 (0.417)	0.254 (0.243)

*p* and r values were calculated by the Pearson test. HU = Hounsfield unit; IT = insertion torque; ISQ = implant stability quotient.

**Table 2:** Mean Hounsfield units, maximum insertion torque, and implant stability quotient values and corresponding correlations with implant dimensions.

Implant Dimensions (mm)	Type	Number of Implants	Mean HU (±SD)	Mean IT (±SD)	Mean ISQ (±SD)	ID-HU r (*p*-Value)	ID-IT r (*p*-Value)	ID-ISQ r (*p*-Value)
4.0 × 10	CMI	4	386 ± 161	35.0 ± 0.0	79.3 ± 1.7	−0.397 (0.002)	−0.163 (0.214)	−0.153 (0.243)
4.1 × 10	SLActive	24	473 ± 161	37.5 ± 6.0	76.4 ± 6.1			
4.5 × 10	CMI	20	409 ± 134	35.5 ± 4.0	75.4 ± 5.6			
4.8 × 10	SLActive	6	277 ± 136	34.2 ± 4.0	76.1 ± 5.4			
5.0 × 10	CMI	6	255 ± 176	35 ± 3.2	74.0 ± 10.3			

*p* and r values were calculated by the Pearson test. HU = Hounsfield unit; IT = insertion torque; ISQ = implant stability quotient; ID = implant dimension.

## Data Availability

Data sharing not applicable. Access to the source dataset is only permitted to employees of Department of Prostondontics, Seoul National University Dental Hospital.
